# RGB-D Data-Based Action Recognition: A Review

**DOI:** 10.3390/s21124246

**Published:** 2021-06-21

**Authors:** Muhammad Bilal Shaikh, Douglas Chai

**Affiliations:** School of Engineering, Edith Cowan University, Perth, WA 6027, Australia; d.chai@ecu.edu.au

**Keywords:** action recognition, deep learning, data fusion, RGB-D

## Abstract

Classification of human actions is an ongoing research problem in computer vision. This review is aimed to scope current literature on data fusion and action recognition techniques and to identify gaps and future research direction. Success in producing cost-effective and portable vision-based sensors has dramatically increased the number and size of datasets. The increase in the number of action recognition datasets intersects with advances in deep learning architectures and computational support, both of which offer significant research opportunities. Naturally, each action-data modality—such as RGB, depth, skeleton, and infrared (IR)—has distinct characteristics; therefore, it is important to exploit the value of each modality for better action recognition. In this paper, we focus solely on data fusion and recognition techniques in the context of vision with an RGB-D perspective. We conclude by discussing research challenges, emerging trends, and possible future research directions.

## 1. Introduction

Human action recognition (HAR) has recently gained increasing attention from computer vision researchers with applications in robot vision, multimedia content search, video surveillance, and motion tracking systems. The recent developments in artificial intelligence have stimulated computer vision researchers to investigate problems in recognizing actions. Coupled with the vast amount of digital data available today, the rise of deep learning has resulted in a dramatic increase in computing resources and offers attractive opportunities for designing efficient action recognition systems.

The development of low-cost sensors such as Microsoft Kinect [1], Intel RealSense [2], and Orbbec [3] has sparked further research into action recognition. These sensors collect data in various modalities such as RGB video, depth, skeleton, and IR. All these modalities have their own characteristics that can help answer challenges related to action data and provide potential opportunities for computer vision researchers to examine vision data from different perspectives.

Herath et al. [4] have defined action as “the most elementary human-surrounding interaction with a meaning”. HAR is the process of labeling the actions performed by humans within a given sequence of images, where it becomes the classification of goals of a human agent in a series of image frames. Action recognition typically aims to discover the class of short, segmented, atomic action. However, action detection (or event detection, or annotation) algorithms reason not only about whether an action occurs somewhere in a video, but also on the temporal extent of when it occurs. Due to their multifaceted nature, some of these approaches refer to action recognition as plan recognition, goal recognition, intent recognition, behavior recognition, location estimation, event recognition, action recognition, and interaction recognition. Some of the terms referenced in the literature in relation to action are defined in Table 1.

Early research on Human Action Recognition was dominated by the analysis of still images or videos [5,6,7,8,9,10], localizing the actor in a video spatio-temporally using bounding boxes, temporal extent, and a spatio-temporal cuboid which contains a particular action. Action recognition remains challenging due to problems posed by background clutter, partial occlusion, viewpoint, lighting changes, execution rate, and biometric variation. These challenges remain even with the application of current deep learning-based approaches [4,11]. Understanding information from images is a challenging process that has engaged thousands of researchers for over four decades and studies are still far from developing a general-purpose machine that can “see” [12].

Human Action Recognition has many applications, including the automated annotation of user videos, indexing and retrieving user videos, automated surveillance, monitoring elderly patients using specially adapted cameras, robot operations, and live blogging of actions. In recent times, the availability of massive amounts of video data has provided significance to the understanding of video data (through a sequence of images) with the possibility of solving problems such as scene identification, searching through video content, and interaction recognition through video scenes [13].

Several survey papers [14,15,16,17,18,19,20,21,22,23,24,25] have discussed action recognition from different perspectives. This survey is unique as it focuses action recognition methods on various RGB-D modalities. RGB-D, which stands for Red Green Blue-Depth, provides depth information associated with corresponding RGB data. However, some relatable surveys, such as those in [26,27,28,29], study from a single modality perspective or compare the characteristics of different datasets. According to our knowledge, there is no review with a focus specifically on data fusion and vision-based action recognition in the context of RGB-D data. The literature was searched by using the keywords “action recognition” over the period from 2010 to 2020. In vision-based action recognition, classification techniques take distinctive characteristics from each modality and apply computer vision methods. This paper offers computer vision researchers a set of potential opportunities to explore vision data by exploiting the natural characteristics of different modalities.

A novel contribution of this review is the focus on RGB-D data-based action recognition using deep learning-based methods. Moreover, this work distinguishes itself from other studies through the following contributions.

1. Review of state-of-the-art action recognition techniques on common RGB-D datasets that will provide readers with an overview of recent developments in action recognition.
2. Analysis of current methods from a perspective of multimodality and hybrid classification methods.
3. Intuitive categorization and analysis of recent and advanced classical machine learning-based and deep learning-based techniques.
4. Discussion of the challenges of data fusion and action recognition and potentials future research directions.

**Figure 1:**
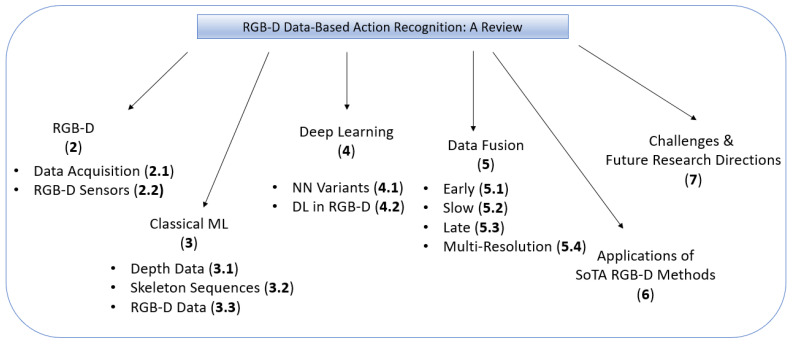
Structure of this paper. Numbers in brackets refer to the section numbering.

The remainder of this paper is organized as follows. Section 2 discusses RGB-D modality and data acquisition. Section 3 and Section 4 review the classical machine learning-based methods and deep learning-based methods, respectively. Section 5 discusses the use of different data fusion techniques used in HAR. Section 6 gives a summary of applications of state-of-the-art RGB-D methods in different scenarios. Section 7 outlines different challenges in data fusion and action recognition techniques, and discusses the future research directions. Finally, Section 8 concludes the review. The hierarchical structure of this paper is shown in Figure 1.

## 2. RGB-D

**Figure 2:**
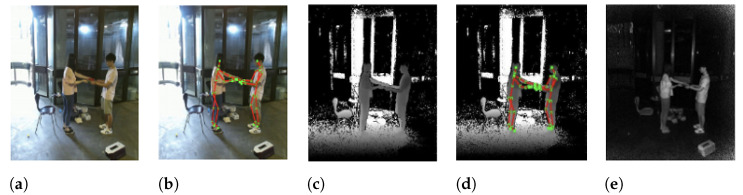
Example data captured by an RGB-D sensor as taken from the NTU RGB-D dataset [31] in (**a**) RGB, (**b**) RGB + Skeleton Joints, (**c**) Depth, (**d**) Depth + Skeleton Joints, and (**e**) IR modalities.

RGB-D generally refers to Red, Green, Blue plus Depth data captured by RGB-D sensors. An RGB-D image provides a per-pixel depth information aligned with corresponding image pixels. An image formed through depth information is an image channel in which each pixel relates to a distance between the image plane and the corresponding object in the RGB image. The addition of depth information to conventional RGB image helps improve the accuracy and the denseness of the data. An example of data captured by an RGB-D sensor is shown in Figure 2. RGB-D data acquisition and different consumer preferred sensors will be discussed in following subsections.

### 2.1. RGB-D Data Acquisition

Acquisition of depth information is mainly based on triangulation and Time-of-Flight (ToF) techniques. The former technique may be implemented passively using stereovision, which retrieves depth information by capturing the same scene from different point of views. Stereovision emulates a human vision principle where depth is computed as a disparity between images taken from different viewpoints. This may require knowledge of the geometry of cameras and calibration needs to be performed for each change in system configuration. An active approach relies on structured light, which uses an IR light pattern onto the scene to estimate disparity through varying object’s depth. In addition to this, ToF and Light Detection and Ranging (LiDAR) scanners measure the time that light takes to hit an object’s surface and return to the detector. LiDAR uses mechanical components to its surrounding. However, ToF performs distance computation using integrated circuits. Chen et al. [22] and others [30] have briefly surveyed depth data acquisition in RGB-D sensors.

### 2.2. RGB-D Sensors

Most of the consumer RGB-D sensors rely on structured light or ToF approaches. Such RGB-D sensors possess noise and data distortions, which are tackled by specifically designed algorithms. Nevertheless, ToF provides a better depth resolution than the others, which is about a few millimeters. Moreover, structured light systems are not beneficial in outdoor scenarios because solar light strongly affects IR cameras. HAR tasks that do not require very high depth resolution and precision have been easily implemented using both structured light sensors and ToF devices. Such devices represented a very good compromise between cost, performance, and usability, and allowed implementation of unobtrusive and privacy-preserving solutions. Some consumer-preferred RGB-D sensors are outlined in the following subsections.

#### 2.2.1. Microsoft^®^ Kinect™ Sensors

**Figure 3:**
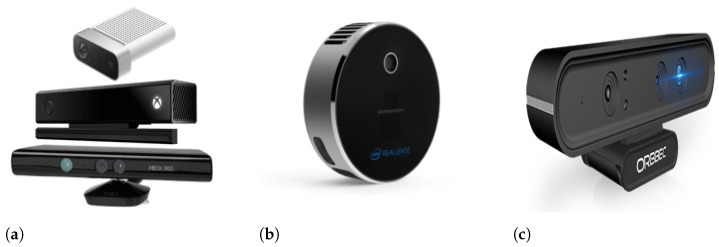
Various RGB-D sensors: (a) Microsoft Kinect [33,34,35], (b) Intel RealSense L515 [36], and (c) Orbbec Astra Pro [37].

Microsoft released the Kinect RGB-D sensor, a low-cost but high-resolution tool that could be easily interfaced to a computer, and whose signals could be easily manipulated through common academic practices. The Kinect sensor V1 uses structured light, and Kinect V2 is based on ToF. The latter exhibits less software complexity but requires fast hardware, such as pulse width modulation (PWM) drivers. The Kinect technology pushed the development of depth-based algorithms and processing approaches. Kinect has been discontinued, but alternative sensors are available in the market. Azure Kinect is a recent spatial computing developer kit with computer vision and speech models, and a range of development interfaces that can be connected to Azure cognitive services. Azure Kinect is not available for consumers and thus not a replacement of Kinect. Michal et al. [32] presented a comprehensive evaluation of Azure Kinect and its comparison with both versions of Kinect. Different versions of Kinect Sensor are shown in Figure 3a (from bottom to top—Kinect v1, Kinect v2, and Azure Kinect).

The Kinect sensor makes the task of capturing RGB-D data easier by sensing the depth dimension of the subject and its environment. It also interprets the movement performed by a subject and transforms it into a format that practitioners can use for new experiments. Computer vision researchers have leveraged Kinect’s vision technology for performing tasks such as aiding children to overcome autism [38] and for doctors in their operating rooms. Azure Kinect has been released for developers and industries which will potentially transform human–computer interaction in various industries including manufacturing, education [39], healthcare [40], retail [41], transportation [42], and beyond.

#### 2.2.2. Intel^®^ RealSense™ Depth Cameras

Intel RealSense depth cameras encompass a family of stereoscopic and portable RGB-D sensors which includes subpixel disparity accuracy, assisted illumination, and performs well even in outdoor settings. Keselman et al. [43] provided a brief overview of Intel RealSense cameras. The R400 family is successor to the R200 family that includes improvements in its stereoscopic matching algorithm and correlation cost function as well as an optimization in design, which enables the R400 family to consume lower power than R200 while operating on the same image resolutions. Intel has divided its RGB-D sensors into different categories which includes stereo depth, LiDAR, coded light, and tracking sensors. The Intel RealSense LiDAR Camera L515 [44] shown in Figure 3b is the smallest high-resolution LiDAR depth camera to date. The Intel D400 [45] series uses Active IR stereo technology. The Intel SR [46] series uses coded light technology; however, the recently introduced L series uses LiDAR technology for acquiring depth information. The L series has significantly reduced the size of the sensor, which can accelerate the use of RGB-D sensors in HAR.

#### 2.2.3. Orbbec^®^ Depth Cameras

Orbbec Astra sensors incorporate processor which replaces traditional cable-based connection to sensor. Similar to Kinect, the Orbbec Astra Pro device as shown in Figure 2 includes an RGB camera, a depth camera, an IR projector, and two microphones. In addition to this, the Orbecc camera-computer package is economical compared to Kinect or RealSense devices. Several SDKs are available including Astra SDK (developed by the manufacturers of the sensor) and OpenNI framework for 3D natural interaction sensors. The use of different sensors in the same problem could affect the accuracy of the process. Coroiu et al. [47] demonstrated safe exchange of Kinect sensor with the Orbbec sensor. According to the experiments, over 16 classifiers demonstrated that choice of sensor does not affect the accuracy. However, seven classifiers produced a drop-in accuracy. Furthermore, calibration algorithms using different RGB-D sensor are compared in [48]. In general, RGB-D sensors exhibit acceptable accuracy, but in some cases, calibration processes are critical to increase the sensor’s accuracy and enable it to meet the requirements of such kinds of applications.

## 3. Classical Machine Learning-Based Techniques

**Figure 4:**
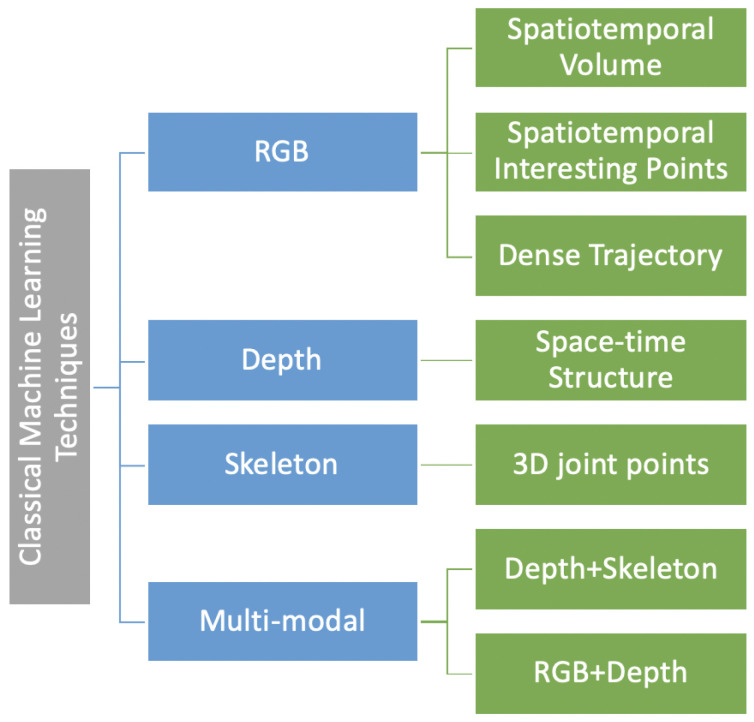
Hierarchy of action recognition techniques based on handcrafted features that use classical machine learning.

Classical machine learning-based action recognition techniques use handcrafted features and can be classified on the basis of RGB data [18], depth data [49,50], skeleton sequences [51], and methods using a combination [52] of these data modalities (as illustrated in Figure 4). Table 2 summarizes the best performing techniques which achieved benchmark accuracies for popular RGB-D datasets in action recognition research. The following subsections will discuss depth-, skeleton-, and RGB-D-based methods.

### 3.1. Depth Data-Based Techniques

Motion changes in the depth maps of the human body are used to represent action. Depth data can be observed as a space–time structure which is extracted from the appearance and motion information to describe human actions. Yang et al. [53] have proposed a supernormal vector feature through a depth map sequence for action representation. Oreifej et al. [49] have proposed an orientation histogram feature of 4D normal vectors to represent the appearance information of a 3D spatio-temporal depth structure. Rehmani et al. [54] have proposed the idea of the main direction of a depth-curved surface where a perspective-independent feature and a principal component histogram are used to represent action. Yang et al. [55] have proposed the Depth Motion Map (DMM) to project spatio-temporal depth structure onto motion history maps. More recent motion history maps are represented by Histogram of Gradients (HoG) features in series to represent actions. Chen et al. [56] have used local binary features instead of HoG features; they [57] also investigated spatio-temporal depth structure from front, side, and upper directions. Miao et al. [58] have considered discrete cosine variation to compress the depth map and represent action through features using transform coefficients.

### 3.2. Skeleton Sequence-Based Techniques

Changes in position and appearance changes in human joint points between frames are used to describe action. Xia et al. [59] have modeled action through a discrete hidden Markov model. Action features have also been extracted through 3D Histograms of Oriented Displacements (HoD) [60], Accumulation of Motion Energy (AME) function aided with the Eigenjoint-based method [51], and through a longest common sequence algorithm [61] to select high-discriminative power features from the relative motion trajectories of the skeleton.

### 3.3. RGB-D Data-Based Techniques

The research results in [50,54,62] show that depth-based methods achieve better action recognition performance than RGB-based methods. Therefore, some researchers have also tried a fusion of different modalities. Chaaroui et al. [63] have investigated the fusion of skeleton and depth data to overcome problems caused by occlusion and perspective changes in skeleton features. In addition, a sparse regression learning-based method to fuse depth and skeleton features has been proposed by Li et al. [52]. A multi-kernel-based learning method for describing actions has been proposed by Althloothi et al. [64]; they calculated spherical harmonics through depth data and fused this with the spatial information of skeleton joints. Furthermore, RGB and depth data fusion has also been attempted by some researchers. For example, Liu et al. [65] used generic algorithms, Jalal et al. [66] merged spatio-temporal features, and Ni et al. [67] introduced the multi-level fusion of RGB and depth data features. However, to answer the missing modality problem, Kong et al. [68] have proposed a discriminative relational representation learning (DRRL) method. In the absence of a single modality in testing, this method transfers knowledge from training data to substitute the missing modality and achieves better recognition performance. The main concern with RGB-D data fusion is that it adds more computational complexity to the action recognition algorithm. Yu et al. [69] have proposed a binary representation for RGB-D data fusion with structure-preserving projections. This approach produced high efficiency and effectiveness on various action recognition benchmarks of RGB-D data. Different challenges associated with RGB-D data fusion techniques are discussed in Section 7.1.

## 4. Deep Learning

Computer vision researchers have directed considerable attention to the application of deep learning in action recognition. The classical machine learning-based methods are based on handcrafted features, which are not robust. Deep learning-based methods have been utilized due to their automated feature learning from images. Researchers have extracted action features from RGB data, depth data, and skeleton sequences using deep learning methods. The following subsections discuss the fundamental variants of neural networks, and later we present some modern deep learning-based approaches used in RGB-D data.

### 4.1. Neural Networks Variants

Recent successes in deep neural networks have boosted research in pattern recognition and computer vision. Some commonly used variants of neural networks are briefly outlined in following subsections.

#### 4.1.1. Convolutional Neural Networks (CNN)

**Figure 5:**
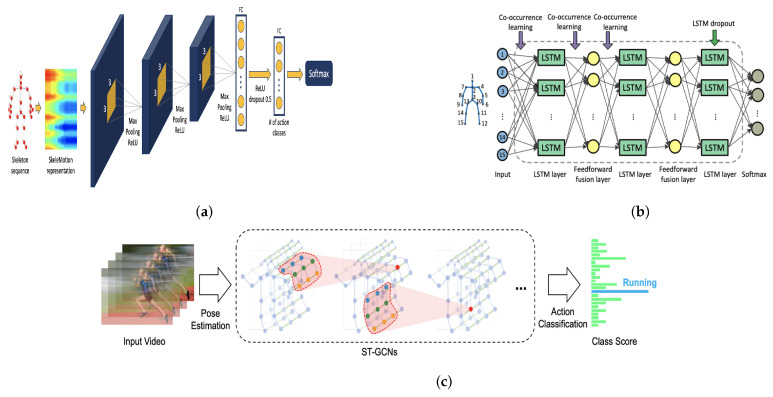
Illustration of deep learning techniques for processing RGB-D data. (**a**) Convolutional Neural Network (CNN). (**b**) Long Short-Term Memory (LSTM). (**c**) Graph Convolutional Network (GCN).

CNNs represent one of the most notable deep learning approaches, where they have been highly effective in a variety of computer vision applications. CNNs are good at recognizing patterns in Euclidean data, i.e., images, text, and videos. CNN works with a mathematical function called convolution, which is a special kind of linear operation. In convolution, input neurons are multiplied with a set of weights that are commonly known as filters or kernels. The filters act as a sliding window across the whole image and enable CNNs to learn features from neighboring cells. Within the same layer, the same filter will be used throughout the image, this is referred to as weight sharing. For example, using a CNN to classify images of dogs vs. non-dogs, the same filter could be used in the same layer to detect the nose and the ears of the cat. There are four basic ideas behind CNN that benefit from the characteristics of natural signals: local connections, shared weights, pooling, and the use of many layers [70]. These four key ideas can be labeled as the Convolution layer, Rectified Linear Unit (ReLU) layer, Pooling, and Fully Connected (FC) Layer, respectively. An example of CNN architecture is presented in Figure 5a, originally shown in [71].

#### 4.1.2. Recurrent Neural Networks (RNN)

The Recurrent Neural Network (RNN), Auto-Associative, or Feedback Network is a type of neural network that has variants including Gated Recurrent units. RNNs have been quite successful in conducting tasks like speech recognition, caption generation, machine translation, image/video classification, human dynamics, and action recognition, among other applications. The RNN function is an alternative to CNN; the RNN function is good at learning dependencies among spatially correlated data like image pixels [72]. RNN cannot store information for a longer duration. Long Short-Term Memory (LSTM) is a special kind of RNN capable of learning temporal relationships on a long-term scale. LSTM [73] uses a gates mechanism: write (input gate), read (output gate), and reset (forget gate), where this gates mechanism controls the behavior of its memory cells. The use of LSTM has produced effective results in speech recognition, especially in phoneme recognition. However, learning with LSTM is often challenging in real-time sequences [74]. An example of LSTM architecture is presented in Figure 5b, originally shown in [75].

#### 4.1.3. Graph Convolutional Networks (GCN)

Earlier variants of neural networks are implemented using regular or Euclidean data. However, real-life data have a graph structure that is non-Euclidean. Therefore, the non-regularity of data structures has led to advancements in graph neural networks. Graph Convolutional Networks (GCN) are also considered as one of the basic Graph Neural Networks variants. Convolution in GCNs is the same operation as in CNNs. GCNs [76] are an efficient variant of CNNs on graphs. In GCNs, the model learns from the neighboring nodes by stacking layers of learned first-order spectral filters followed by a nonlinear activation function to learn graph representations. For simplicity, GCNs take a graph with some labeled nodes as input and generate label predictions for all graph nodes. GCNs could be divided into two approaches: Spatial GCNs and Spectral GCNs. An example of GCN is presented in Figure 5c, originally shown in [77].

### 4.2. Deep Learning-Based Techniques Using RGB-D Data

Deep learning can directly obtain hierarchical features from different data modalities and provides a more effective solution. Accordingly, appearance and optical sequences can be used as inputs to deep networks. Besides aspects of appearance and motion information, deep learning-based methods can also be applied using depth sequences and skeleton joint information. Wang et al. [102] have used convolution to learn action features from depth data. They [103] combined motion and structure information in a depth sequence by pairing structured dynamic images at the body, part, and joint levels through bidirectional rank pooling. Every pair is constructed from depth maps at each granularity level and serves as input to CNN. Song et al. [104] have proposed a model that uses different levels of attention in addition to an RNN with LSTM to learn discriminative skeleton joints. Ye et al. [105] have embedded temporal information with dense motion trajectories to learn actions.

Yan et al. [71] have modeled relationships between graphs and joints by using a graph-oriented CNN. Deep learning-based feature learning has been shown to provide better performance than handcrafted feature extraction methods; however, there are still challenges concerning RGB-D data fusion. Deep learning-based action recognition methods use different standalone as well as hybrid neural network architectures, which can be classified as Single-Stream, Two-Stream, Long-term Recurrent Convolutional Network (LRCN), and Hybrid network-based architectures. The following subsections summarize these architectural styles.

#### 4.2.1. Single Stream

A single-stream model is similar to the AlexNet [106] type of image classification network. Single-stream architecture can take advantage of regularization through local filters, parameter sharing at convolution layers, and local invariance building neurons (max pooling). Such neural network architecture shifts the engineering focus from feature design strategies to network structure and hyperparameter tuning strategies. Architectural details from AlexNet [106] can be used with different hyperparameter configurations. A single-stream architecture fuses information from all the frames in the softmax layer connected to the last fully connected layers with dense connections. Given an entire action video, the video-level prediction can be produced by forward propagation of each frame individually through the network and then averaging individual frame predictions over the duration of the video. However, single-stream architecture has been a foundation for other extended architectures. Some possible extensions to single-stream architecture have been explored by Baccouche et al. [107], Ji et al. [108], and Karpathy et al. [86].

#### 4.2.2. Two Stream

The two-stream model uses two disjointed CNNs containing spatial and temporal information, which are later fused together. The spatial network performs action recognition from single video frames, while the temporal network learns to recognize action from motion, i.e., dense optical flow. The idea behind this two-stream model relates to the fact that the human visual cortex contains two pathways for object and motion recognition, i.e., the ventral stream performs object recognition and the dorsal stream recognizes motion. Spatial-stream CNN is modelled similar to the single-frame model discussed earlier. Given an action video, each frame is individually passed through the spatial network where an action label is assigned to each frame. The temporal-stream CNN is not the same as motion-aware CNN models (which use stacked single video frames as input). It takes stacked optical flow displacement fields between several consecutive frames as input to explicitly learn a temporal feature.

In two-stream models, the pioneering work of Simonyan and Zisserman [109] uses a single image and multi-optical flow sequence stack as input to the 2D CNN. Zhang et al. [110] have extended Simonyan and Zisserman’s [109] work by using a motion vector instead of optical flow as an input to improve performance and comprehend real-time action recognition. Feichtenhoer et al. [111] have proposed an innovative approach involving moving the classification layer to the middle of the network for spatio-temporal information fusion, and this was shown to improve the accuracy. Wang et al. [112] have contributed to the input and training strategy of convolution and proposed Temporal Segment Network (TSN), improving the two-stream CNN. The notion of TSN was based on long-range temporal structural modeling. Later, Lan [113] and Zhou [114] enhanced the TSN. Carreira et al. [115] adopted the structure of Inception-v1 and inflated two-stream CNN to 3D CNN for action recognition. Zhu et al. [116] have expanded two-stream CNN to a 3D structure by drawing out the pooling operation.

#### 4.2.3. Long-Term Recurrent Convolutional Network (LRCN)

**Figure 6:**
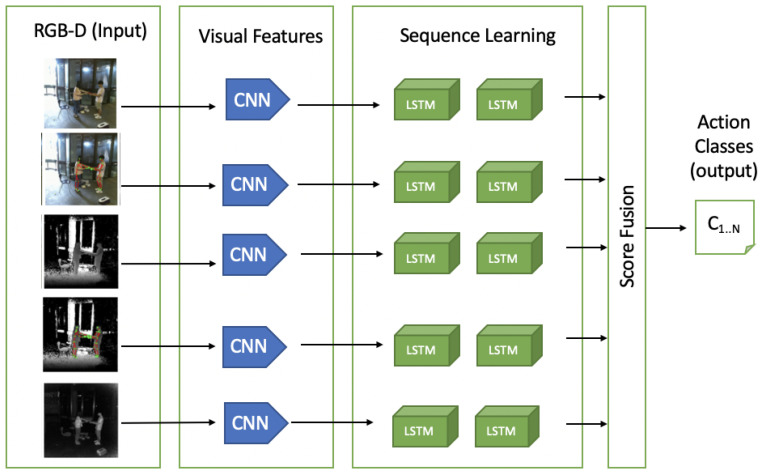
A possible architecture of LRCN with RGB-D input. Input from each modality, i.e., RGB, RGB + Skeleton joints, Depth, Depth + Skeleton joints, and IR are passed through a CNN layer for extracting visual features and an LSTM layer for sequence learning. Scores from each model are then fused and mapped to the number of classes for predictions. Visuals of RGB-D input are taken from NTU RGB-D 60 dataset [90].

LRCN uses CNN in co-ordination with an LSTM-based network. In LSTM-based deep learning methods, actions can be represented as feature changes between frames in the video, LSTM is widely used to improve action recognition techniques. Ng et al. [117] have presented a linear RNN for recognizing human actions that connects the output of a CNN with an LSTM cell. A new architecture—P3D ResNet—has been proposed by Qiu et al. [118], which uniquely places all the variants of blocks in a different placement of ResNet. In skeletal data, to deal with noise, Liu et al. [119] extended the idea to analyze spatio-temporal domains simultaneously by introducing an effective tree structure-based traversal framework. This framework uses a cross-modal feature fusion strategy within LSTM unit and a gating mechanism to learn the reliability of sequential data in long-term context representation. For mapping video frames with variable length inputs to variable length outputs, Donahue et al. [120] have proposed an LRCN. Unlike those methods that learn CNN filters based on a stack of a fixed number of input frames, LRCN [120] is not constrained to fixed-length input frames and thus can learn to recognize more complex action video. As illustrated in Figure 6 (RGB-D input visuals taken from the work in [31]), individual video frames are first passed through CNN models with shared parameters and are then connected to a single-layer LSTM network. More precisely, the LRCN model combines a deep hierarchical visual-feature extractor, i.e., a CNN feature extractor, with an LSTM that can learn to recognize temporal variations in an end-to-end fashion.

#### 4.2.4. Hybrid Deep Learning-Based Techniques for HAR

Hybrid deep learning-based approaches that use advanced variants of deep learning architectures to produce state-of-the-art action recognition accuracy on popular datasets are discussed below.

On a large-scale YT-8M dataset, Abu-El-Haija et al. [121] pretrained an LSTM network and discovered that pretraining on a large-scale data set generalizes well on datasets such as Sports-1M [86] and ActivityNet [122]. However, Jinyoung et al. [123] proposed an extensible hierarchical method for detecting generic interactive actions (by combining spatial relations with movements between two objects) and inherited actions determined through an ontology of rule-based methodology. This technique outperforms other techniques on the ActionNet-VE dataset [124].

Yuan et al. [125] have proposed the Multi-Granularity Generator (MGG), which produces temporal action proposals through two producers: the Segment Proposal Producer (SPP) and the Frame Actionness Producer (FAP). SPP generates a segment proposal from a coarse perspective, while FAP produces a finer actionness evaluation for each video frame. Both producers are combined to reflect two different granularities. MGG can be trained in an end-to-end fashion and performs better than state-of-the-art methods on the THUMOS-14 dataset. In addition, Zhaofan et al. [126] have provided a novel neural network architecture that uses spatio-temporal representation learning by Local and Global Diffusion (LGD) in parallel. This architecture is composed of LGD blocks, which update local and holistic features. In addition to this, a kernelized classifier is used for video recognition.

An efficient and generic Temporal Shift Module (TSM) was proposed by Lin et al. [127] which claims the performance of 3D CNN while maintaining the complexity of 2D CNN. TSM facilitates information sharing among neighboring frames by shifting a portion of the channels along the temporal dimension. TSM achieves state-of-the-art results on the Something-Something dataset. However, Zhu et al. [88] have initiated the idea of an Action Machine which is a simple fast method extended from an Inflated 3D CNN by adding a module of 2D CNN and pose estimation. The Action Machine takes input that is cropped through person-bounding boxes, fusing predictions from RGB images and poses. Action Machine produced state-of-the-art results on NTU RGB-D datasets and competitive results on smaller datasets of Northwestern-UCLA, MSR-DailyActivity3D, and UTD-MHAD [83,91].

Girdhar et al. [128] have proposed the Video Action Transformer Network (VATN) which uses transformer-style architecture that aggregates features from the spatio-temporal context. VATN uses an attention mechanism that learns to emphasize hands and faces. On the Atomic Visual Actions (AVA) dataset, VATN outperformed state-of-the-art methods by using raw RGB frames as input. Moreover, Hu et al. [129] have explored the modality-temporal mutual information to learn time-varying information and cross-modal features jointly. They introduced an action feature called a modality-temporal cube, which characterizes RGB-D actions from a comprehensive perspective. Their proposed framework uses deep bilinear blocks that pool input from both modality and temporal directions.

On the other hand, the concept of the Gate Shift Module (GSM) in the spatio-temporal decomposition of 3D kernels was introduced by Sudhakaran et al. [130]. GSM is added to a 2D CNN that learns route features and combines them with less computational complexity and additional parameters overhead. This technique achieves state-of-the-art results on the Something-Something-v1 dataset and competitive results on other datasets. In addition, Caetano et al. [77] have proposed the Skelemotion, which extends Spatio-Temporal Graph Convolutional Networks (ST-GCN) by introducing a Graph Vertex Feature Encoder (GVFE) and Dilated Hierarchical Temporal Convolutional Network (DH-TCN). GVFE learns vertex features by encoding raw skeleton features data, while DH-TCN captures both short- and long-term dependencies. This architecture uses fewer layers and parameters, and it competes better with state-of-the-art methods on NTU RGB-D 60 and NTU RGB-D 120 datasets.

Korbar et al. [85] have introduced a clip sampling scheme that selects salient temporal clips within a long video. This technique improves the state-of-the-art and reduces computational costs significantly. However, Wang et al. [96] have proposed Channel-Separated Convolutional Networks (CSNN) which demonstrate the benefits of factorizing 3D convolutions by separating spatio-temporal interactions and channel interactions. The latter is a form of regularization that improves accuracy and lowers computational costs.

In [87], Object-Related Human Action recognition through Graph Convolution Networks (OHA-GCN) was proposed which constructs graphs using selective sampling of human and object poses. OHA-GCN late fuses class scores from human poses and object pose streams for action classification. Furthermore, Wang et al. [97] have proposed a network that creates improved Dense Trajectories (iDT) descriptors and i3D optical flow features with CNNs, thus reviving classical handcrafted representations.

Liu et al. [131] have introduced a novel neural network called CPNet that learns evolving 2D fields with temporal consistency. CPNet achieved state-of-the-art results on both Jester [132] and Something-Something datasets [127,130]. Moreover, Martin et al. [133] have introduced the novel approach for fine-grained categorization of driver behavior. They focused on key challenges such as recognition of fine-grained behavior inside the vehicle cabin, focusing on diverse data streams and a cross-view recognition benchmark and adopting prominent methods for video and body pose-based action recognition to provide challenging benchmarks. Besides, Munro and Damen [134] have exploited the correspondence of modalities as a self-supervised alignment approach in addition to adversarial alignment, which outperforms other unsupervised domain adaptation methods.

Table 3 summarizes the key differences among deep learning-based action recognition methods that have evolved over the last decade. It can be observed that most of the techniques are not applied on RGB-D datasets. Accuracy is the most preferred metric for action recognition, where sometimes these techniques benefit from the use of extra training data.

In HAR, the implementation and execution of deep learning-based methods can often be time-consuming. Experimental platforms provide abstraction, customization, community, and advanced hardware-level support. This is important for the development of robust and flexible deep learning-based action recognition systems. Some platforms are intuitive and highly abstract, but such abstractions or wrappers can make it difficult to debug or apply explicit changes to algorithms at low levels. As performance demand relies on high-end hardware and multiple graphical processing units (GPU), support is a must when experimenting with big data-related problems.

## 5. Data Fusion Techniques

Data fusion supports diversity, which enhances the uses, advantages, and analysis of ways that cannot be achieved through a single modality. Fusion techniques can be deployed at different stages in the action recognition process to acquire combinations of distinct information. The following are some popular ways of fusing action data in RGB-D datasets.

### 5.1. Early Fusion

**Figure 7:**
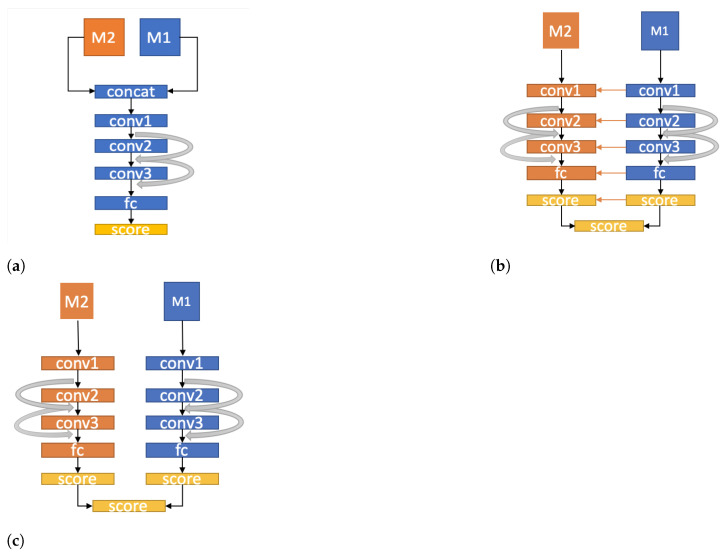
An example of (**a**) early, (**b**) slow, and (**c**) late fusion in HAR. Note that the input modalities are not limited in the above two modalities.

The early fusion approach captures information and combines it across a raw or data level. To achieve this, the filters on the first layer in the neural network are modified. This direct and early connectivity of the raw data helps the network to detect the fused feature vectors at an early stage. For an entire action data sequence, some randomly selected sample instances are often passed through the system, and their class predictions are then averaged to produce action class predictions, as illustrated in Figure 7a (adapted from the work in [135]).

### 5.2. Slow Fusion

The slow fusion approach fuses the features extracted from raw data throughout the neural network so that the higher layers have access to more global information. This is achieved by performing a CNN-, RNN-, or LSTM-based operation to calculate the weights and extend the connectivity of all layers. For example, as shown in Figure 7b, raw data from two-different modalities are filtered in the first convolution layer. The next layers iterate this process in the network with different filter configurations. Therefore, the information across all the input data can be assessed by the subsequent convolution layers. Given an entire human action data sequence, action classification is performed over the entire dataset through the network and then averaging individual predictions throughout the action sequence.

### 5.3. Late Fusion

The late fusion approach combines the action information at the deepest layers in the network, for example, a HAR architecture network consisting of two separate CNN-based networks with shared parameters up to the last convolution layer. The outputs of the last convolution layer of these two separate network streams are processed to the fully connected layer. Global action characteristics are fused, and the classification score is then averaged or concatenated by different holistic operations at the score layer. This approach has been relatively successful in most of the HAR systems. An illustration of late fusion is shown in Figure 7c.

### 5.4. Multi-Resolution

In order to speed up the above-mentioned models while retaining their accuracy, a multi-resolution architecture has been proposed by Karpathy et al. [86]. The multi-resolution model consists of two separate networks (fovea and cortex networks) over two spatial resolutions. The architectures of fovea and cortex networks are similar to the single-frame architecture. However, instead of accepting the original input, these networks record reduced sized inputs. More precisely, the input to the fovea model is the center region at the original spatial resolution. In contrast, for the context stream, the downsampled frames at half the original resolution are used. The total dimensionality of the inputs is, therefore, halved. Moreover, the last pooling layer is removed from both the fovea and cortex networks. The activation outputs of both networks are concatenated and fed into the first fully connected layer.

All the above-mentioned network variations were trained on the Sports-1M dataset [86], which consists of 200,000 test videos. The results showed that the variation among different CNN architectures (e.g., Single Frame, Multi-Resolution, Early, Late, and Slow Fusion) was surprisingly insignificant. Furthermore, the results were significantly worse than the state-of-the-art handcrafted shallow models. This may be because these models cannot capture the motion information in many cases. For example, the slow fusion model is expected to implicitly learn the spatio-temporal features in its first layers, which is a difficult task. To resolve this issue, a two-stream CNN model was proposed by Simnonyan and Zisserman [109] to explicitly take into account both spatial and temporal information in a single end-to-end learning framework.

## 6. Applications of State-of-the-Art RGB-D Methods

RGB-D based Human action recognition (HAR) is a widely studied computer vision problem. As the imaging technique advances and the camera device upgrades, novel approaches for HAR constantly emerge. Some significant application areas are discussed below.

### 6.1. Content-Based Video Summarization

At present, the enormous use of multimedia devices has given growth to video content. The manual task of video content retrieval is time-consuming and tedious. The authors of [136] used color and texture features to demonstrate the benefit of different feature combinations for video summarization. The work in [137] proposed a real-time video summary by using a threshold based on the probability distribution. Identical features were removed by using redundancy elimination techniques.

### 6.2. Education and Learning

Classifying human actions from RGB-D data plays an important role in education and learning. The exploration of human actions based on visual data in educational institutions can help in recognition and predetermined monitoring of attendance during class. During this process, the instructor-led attendance is time-consuming and requires rigorous observation from the instructor.

With the recent advances in technology, the automated attendance system can now be deployed in a classroom environment. To monitor student attendance, visual data are acquired to register students when they enter or leave the classroom. In [138], the system recognizes students and their activities such as entering and leaving the classroom. The system performs student identification by performing face recognition and motion analysis and performs action recognition to recognize students’ actions. A combination of techniques using RGB-D data can be applied in different education and learning environments for efficient action recognition systems.

### 6.3. Healthcare Systems

The use of action recognition techniques using RGB-Data can benefit healthcare systems. For example, as the elderly are susceptible to disease, healthcare for the elderly has been a major concern. Automated monitoring systems are required to recognize actions such as falls and other abnormal behaviors in the elderly. In [139], the authors proposed an approach to depict the behavior of dementia (Alzheimer’s and Parkinson’s) in patients. RNN variants such as Vanilla RNNS, LSTM, and Gated Recurrent Unit (GRU) are used to detect abnormal actions in elderly patients with dementia. Continuous monitoring of temperature, blood pressure, blood glucose, and blood oxygen is performed using different smartphone sensors. A warning is generated by the system in case of abnormal activity in [140].

### 6.4. Entertainment Systems

HAR has been broadly explored to recognize actions in dance moves. Laptev et al. [141] presented the task of recognizing actions using text-based classifier (regulated perceptron). Space-time functions and nonlinear SVMs are used to classify actions from the film script. In addition, Wang et al. [142] classified film actions using 3D CNN. Two modules, namely, coding and a temporal pyramid pooling layer, were used introduced in order to minimize the loss during learning. A feature linkage layer was incorporated to combine motion and appearance information. HAR is also used to detect dance moves using videos. Kumar et al. [143] analyzed the dataset based on classical Indian dance using an AdaBoost classifier with multiple classes and merged characteristics. On the other hand, Castro et al. [144] discovered that visual information in motion-intensive videos is insufficient to efficiently recognize actions. The experimentation is performed using RGB, optical flow and multi-person pose data.

### 6.5. Safety and Surveillance Systems

RGB-D-based HAR techniques can be used to ensure safety in public venues such as railway stations and airports. Action recognition in such an environment is challenging due to a large number of viewpoints. An abnormal activity can be detected even for objects following the same pattern. For example, a train crossing the railway line is considered a normal activity, while a person crossing a railway line is considered as an abnormal activity. In [145], the authors presented an efficient intelligent system for crowded scene using a deep Gaussian mixture model. The multi-layer nonlinear input transformation improves the performance of the network with a few parameters. HAR can also be used to identify abnormal activity recognition in (Unmanned Aerial Vehicle) UAV-based surveillance [146].

### 6.6. Sports

Highlighting key moments in sports videos is difficult for coaches and players to analyze; it can be time-consuming and uninteresting for the audience to watch long games continuously [147]. Recent studies focus on analyzing player movement individually and in groups for their training and evaluation. Ullah et al. [148] used the pretrained deep CNN model VGG-16 to identify player actions. A deep autoencoder is used to learn changes over time and human actions are classified using SVM. In group activities, graph-based are widely used. Qi et al. [149] classified sports videos using a semantic diagram and an RNN is used to extend the semantic graph model to the temporal dimension.

## 7. Challenges and Future Research Directions

Action recognition remains challenging due to background clutter, partial occlusion, viewpoint, lighting changes, execution rate, and biometric variation. Challenges with data fusion followed by future research directions are discussed below.

### 7.1. Challenges in RGB-D Data Fusion

Deep learning-based techniques in HAR use various data fusion approaches such as early, slow, late fusion, and other variants. Some prominent challenges with data fusion approaches are discussed below.

- RGB-D datasets with different resolutions possesses an inherent challenge in data fusion because each modality has a very different temporal and spatial resolution.
- Practically, individual datasets contain incompatible numbers of data samples, which leads to data size incompatibility. Alignment of modalities to a standard coordinate system for maximizing mutual information sharing is an acute challenge in data fusion.
- Inherently, the information conveyed through each modality has different physical properties, which can be vital for better action learning. Identification of key characteristics from each modality that is contributing towards the overall recognition is an interesting problem.
- Negligible errors produced by RGB-D sensors are often abstracted as noise, which is unavoidable. Balancing noise with other modalities also causes problems in data fusion.
- Most data fusion techniques ignore the noise, but ignoring the noise from datasets collected through different sensors may lead to bias.
- Distinct data modalities confront contradictions, and data inconsistencies may occur. An open challenge is to infer a proper compromise; however, identifying these conflicts, contradictions, and inconsistencies is a fundamental challenge.
- RGB-D sensors may produce spurious data due to environmental or sensor failure issues, which may lead to false inferences based on biased estimations. Therefore, a challenge may arise in predicting and modeling spurious events.
- Other challenging factors include noise, spatial distortions, varying contrast, and arbitrary subject locations in image sequences.

Data fusion techniques vary across different tasks and need to address various challenges in terms of required time and memory management. Other problems may arise depending on the modality under consideration. For example, basic skeleton features (joint coordinates or bone lengths) are used commonly for constructing spatio-temporal graphs. However, offering a high-level description of the human body structure may affect discriminative power for action recognition. However, an innovative strategy for the combining or augmenting of different modalities at an earlier or any later phase of resolution can lead to better data fusion solutions.

### 7.2. Future Research Directions

The discussion and insights drawn from the challenges in different approaches allow us to present several future research directions to develop methods in action recognition. The following research directions may advance the domain.

#### 7.2.1. Combination of Classical Machine Learning and Deep Learning-Based Methods

Classical machine learning approaches have benefited action recognition through redundant and favorable feature extraction. Deep learning-based methods provide autonomous feature engineering and have produced better recognition systems. Designing effective action recognition systems by adding the power of classical machine learning with advanced deep learning-based techniques has some attraction for researchers. For example, Gao et al. [150] proposed a fusion logic of classical machine learning and deep learning-based methods to achieve better performance than single CNN-based pedestrian detector, and it is likely to emerge as an active research area.

#### 7.2.2. Assessment in Practical Scenarios

Most of the RGB-D datasets have been collected in constraint environments. There remains a significant gap between the collected datasets during the last few years and the practical scenario due to insufficient categories, occlusion cases, constrained environment settings, samples, and limited distance variations. Due to these limitations, collected datasets may not substitute the need for outdoor practical scenario-based datasets. Collection and generalization of algorithms over realistic scenarios should gain the attention of researchers.

#### 7.2.3. Self-Learning

Learning labels about individual samples is often overlapping and causes inefficient intra-class similarity. Self-learning action recognition systems can learn from non-labeled training data without any human intervention. Recent advances in deep learning, such as Generative Adversarial Networks (GAN), may improve action recognition systems’ self-learning capability. GAN-based action recognition techniques would be a compelling research direction.

#### 7.2.4. Interpretation of Online Human Actions

Action recognition algorithms focus on well-trimmed segmented data splits. While in an online action recognition system, which aims to observe many mechanisms such as facial expression, visual focus, view angles etc. instantly from a video stream. Interpretation of such human behavior components in online scenarios is an essential step toward more practical and intelligent recognition systems.

#### 7.2.5. Multimodal Fusion

Multimodal data provide richer information than unimodal data. Still, most methods fuse different modalities as separate channels and combine them at a later classification stage without exploiting their corresponding properties in a parallel fashion. Effective use of deep networks for parallel integration of complementary properties from different modalities would a potential research area. Use of multimodal information also helpful in reducing noise from unimodal data. Therefore, integrating multimodal information and incorporating contextual information from the surrounding environment is a way forward for future research. Different fusion schemes are used in various methods for action classification. Thus, future research may devote more attention from researchers to compare these fusion schemes and find the best fusion strategy for action recognition.

## 8. Conclusions

The vision-based recognition of human actions is an important research field in the integrative computer vision and multimedia analytics ecosystem. This review has thoroughly compared and summarized the landscape of vision-based RGB-D sensors. We provided an outline of existing commonly used datasets and highlighted key research that has mainly focused on RGB-D datasets. We also reviewed the latest action recognition techniques that use deep learning in general. We discussed the techniques that have been used over the past decade and divided them into different perspectives. We then presented various available experimental options, along with their characteristics, strengths, and weaknesses, for action recognition researchers.

The results of this paper show that with the availability of low-cost and multi-function sensors, the effects of RGB-D action recognition can be extended to wider application areas. It is evident that deep learning architectures, especially CNN- and LSTM-based methods, have been shown to produce significant results. However, there is a lack of availability of large data sets in different domains. Attention has turned more to RGB, optical flow, and skeletal modalities, so other promising modalities such as depth and IR have not been adequately explored. The challenges are evident with RGB-D action sensors, data sets, recognition, and fusion techniques. Significant efforts are required to address these challenges.

## Figures and Tables

**Table 1:** Terms related to action recognition.

Term	Definition
Gesture, Mime, Sign	Basic movement or positioning of the hand, arm, body, or head that communicates an idea, emotion, etc.
Action, Event	A type of motion performed by a single person during short time period and involves multiple body parts.
Activity	Composed of a sequence of actions.
Interaction	A type of motion performed by two actors; one actor is human while the other may be human or an object.
Unimodal, Single-mode	Having or involving one mode.
Multimodal, Multi-type, Multi-format	Different types of data acquired through sensors.
Fusion, Mixture, Combination	A process for combining different types of sensor data.
RGB-D	Per-pixel depth information aligned with corresponding image pixels.

**Table 2:** Summary of popular action recognition datasets and methods that achieved the best recognition accuracy. Note that PDF stands for probability distribution function, i3D stands for inflated 3D, OF stands for Optical Flow, and GCN stands for Graph Convolutional Networks.

Year	Ref.	Methods (Modality)	Action Datasets	MSR Daily Activity 3D [78]	UT-Kinect [59]	EPIC Kitchen-55 [79]	NW-UCLA [80]	Toyota-SH [81]	HuDaAct [82]	UTD-MHAD [83]	Charades [84]	NTU RGB-D 120 [31]	miniSports [85]	Sports-1M [86]	IRD [87,88]	HMDB-51 [89]	ICVL-4 [87,88]	NTU RGB-D 60 [90]	MSR-Action3D [91]
2012	[92]	2D CNN (RGB-D)							89										
2015	[93]	DTQ-SVM (RGB-D)			100														90
2017	[94]	CNN (RGB-D)		98														75	
2018	[88]	i3D CNN + 2D CNN (RGB-D)								92								94	
2019	[95]	CNN (RGB + OF)									56								
2019	[85]	i3D CNN (RGB)											74						
2019	[96]	3D CNN (RGB)												75					
2019	[87]	GCN (Skeleton)													80		91		
2019	[97]	CNN (RGB)														82			
2019	[98]	TBN-Inception (RGB-Audio + OF)				35													
2020	[99]	3D CNN + GCN (RGB-D)					94	61				86							
2020	[100]	HAMLET (RGB-D)			98					95									
2020	[101]	CNN (RGB-D)					94			92		95						99	

**Table 3:** State-of-the-art action recognition techniques with their key differences. Notations: CA: Code Availability; ET: Extra Training; TL: Transfer Learning; Y: Yes; N: No; mAP: Mean Average Precision; OF: Optical Flow; IR: Infrared; FV: Fisher Vectors; BoW: Bag of Words; iDT: improved Dense Trajectories; E:Early, M:Middle; L:Late.

Ref.	CA	ET	TL	Metric	Network/Classifier	Modality	Fusion	Novelty
[92]	N	N	N	Accuracy	SVM	RGB-D	M	Extracts interest points solely from RGB channels and combines RGB and depth map-based desciptors.
[93]	N	N	N	Accuracy	SVM	RGB-D	L	Modelling of temporal dynamics of human actions by temporal order preserving dynamic quantization method.
[94]	N	N	N	Accuracy	CNN + SVM	RGB-D	M	Deep hierarchical shared-specific defactorization of RGB-D features and a structured sparsity learning machine.
[96]	N	N	Y	Accuracy	3D CNN	RGB	-	Separated spatio-temporal interactions.
[88]	Y	Y	Y	Accuracy	i3D CNN	RGB + Pose	L	Used person cropped frames as inputs.
[95]	N	Y	Y	mAP	CNN	RGB + OF	L	Reformulated neural architecture search for video representation.
[85]	N	-	Y	Accuracy	i3D CNN	IR + OF + RGB	M	Used salient clip sampling to improve efficiency.
[77]	Y	N	N	Accuracy	CNN	Skeleton	E	Employed graph vertex encoding along with few layers and parameters.
[87]	N	N	N	Accuracy	GCN	Skeleton	-	Used human-object related poses.
[97]	N	Y	N	Accuracy	CNN	iDT/FV/BoW	M	Combined classical handcrafted iDT features with CNN extracted features.
[98]	Y	N	Y	Accuracy	3D-CNN + GCN	RGB-D + OF + Audio	M	Architecture for multimodal temporal binding.
[99]	Y	Y	Y	Accuracy	3D-CNN + GCN	RGB-D	M	A spatial embedding with an attention network.
[100]	N	N	Y	Accuracy	CNN	RGB-D	M	Multimodal attention mechanism for disentangling and fusing the salient features.
[101]	N	N	Y	Accuracy	CNN	Skeleton	-	Inflated ResNet coupled with hierarchical classification and iterative pruning.

## Data Availability

Not Applicable.

## Abbreviations

The following abbreviations are used in this manuscript:

AME	Accumulation of Motion Energy
AVA	Atomic Visual Actions
CSNN	Channel Separated Convolutional Networks
CNN	Convolutional Neural Network
DH-TCN	Dilated Hierarchical Temporal Convolutional Network
FAP	Frame Actionness Producer
GPU	Graphics Processing Unit
GVFE	Graph Vertex Feature Encoder
HoD	Histograms of Oriented Displacements
iDT	improved Dense Trajectories
IR	Infrared
HoG	Histogram of Gradients
LGD	Local and Global Diffusion
LRCN	Long-term Recurrent Convolutional Network
LSTM	Long-Short Term Memory
mAP	mean Average Precision
MGG	Multi-Granularity Generator
OHA-GCN	Object-Related Human Action recognition through Graph Convolution Networks
RGB	Red Green Blue
RGB-D	Red Green Blue-Depth
RNN	Recurrent Neural Network
SPP	Segment Proposal Producer
ST-GCN	Spatio-Temporal Graph Convolutional Networks
SVM	Scalar Vector Machines
TSM	Temporal Shift Module
VATN	Video Action Transformer Network
